# Feeding Modalities and the Onset of the Neonatal Abstinence Syndrome

**DOI:** 10.3389/fped.2015.00014

**Published:** 2015-02-26

**Authors:** Anthony Liu, Jaime Juarez, Ajitha Nair, Ralph Nanan

**Affiliations:** ^1^Discipline of Paediatrics, Sydney Medical School – Nepean, The University of Sydney, Penrith, NSW, Australia

**Keywords:** breastfeeding, breast milk, methadone, neonatal abstinence syndrome, feeding modalities

## Abstract

Breast milk has been reported to ameliorate the severity and outcome of neonatal abstinence syndrome (NAS). The mechanism of this beneficial effect of breast milk on NAS remains unclear, as the negligible amount of methadone transmitted via breast milk is unlikely to have an impact on NAS. The aim of this study was to compare the impact of different feeding modalities on the onset of NAS. A retrospective medical record review was conducted on 194 methadone-maintained mother/infant dyads. Infants were categorized on the first 2 days of life as predominantly breastfed, fed expressed human breast milk (EBM), or formula fed. The feeding categories were then analyzed using the onset of NAS as the outcome measure. After adjusting for confounders, there was no significant effect of the modality of feeding on the rates of NAS requiring treatment (*p* = 0.11). Breastfeeding significantly delayed the onset of NAS (*p* = 0.04). The act of breastfeeding in the first 2 days of life had no effect on whether an infant required treatment for NAS when compared to those fed EBM or formula. This only suggests that the advantages of breastfeeding on NAS cannot be substantiated in a small cohort and should not discourage breastfeeding.

## Introduction

Human breast milk has been reported to ameliorate the severity and outcome of neonatal abstinence syndrome (NAS) (1, 2). Prior case reports and small studies have suggested that breastfeeding has a positive effect on NAS in terms of reducing severity of neonatal withdrawal and length of infant hospitalization (3, 4). Methadone concentration in human breast milk was found to be low and was considered to be unlikely to have a pharmacological effect on NAS (5). Thus, the observed beneficial effects of human breast milk on the severity and outcome of NAS are unlikely to be dependent on the pharmacological dose of methadone transmitted in human breast milk.

There is a lack of literature differentiating the effects of the act of breastfeeding from the effects of human breast milk on the severity and outcome of NAS. The aim of this study, therefore, was to compare the effect of different feeding modalities on the onset of NAS. We hypothesized that the breastfeeding process has an independent effect on the severity and outcome of NAS among infants of methadone-maintained mothers.

## Materials and Methods

### Study design

We conducted a retrospective medical record review of infants born to mothers on the methadone maintenance program at two birthing units in Western Sydney between 2000 and 2006 inclusive. History of illicit drug use and smoking was obtained by maternal self-reporting. Polydrug use was defined as the use of two or more different classes of addictive drugs including methadone. In both these centers, mothers on the methadone maintenance program are encouraged to breastfeed their infants unless there are medical or social contraindications (6). Ethics approval was obtained from the Institutional Ethics Committee.

The infants were categorized based on predominant method of feeding during the first 2 days of life. We used the first 2 days as a cut off for our analysis to separate possible confounding factors with regards to increased difficulties with breastfeeding when a neonate undergoes withdrawal, which commonly occurs within 48 h after birth (7). The breastfeeding group included infants having ≥50% of daily feeds as breast-feeds. Infants who had ingested ≥15 ml of expressed human breast milk (EBM), and who were breastfed <3 times per day were categorized into the EBM group. A cut off of 15 ml of EBM ingestion was based on a previous study, which estimated this as the average amount of colostrum ingested during the first day of life (8). Formula fed infants were defined as those having formula for ≥50% of their feeds, and minimal EBM ingestion (<15 ml/day).

### Assessment and treatment of NAS

Infants born to methadone-maintained mothers were observed for signs of withdrawal using the Finnegan objective scoring system (9). Supportive therapy was maintained from birth and pharmacologic treatment with oral morphine for NAS was initiated depending on the Finnegan scores.

Infants were discharged from hospital if there were no signs of withdrawal after 5–7 days; they were feeding well and had no adverse medical conditions or social circumstances. Infants requiring pharmacotherapy for NAS were discharged 1–2 days after the medications were ceased. If the mother wished to discharge her infant early, the infant had to have stable NAS scores and a weaning dose regime was given for administration at home with appropriate follow-up as an outpatient.

### Statistical analysis

Statistical analyses were performed using statistical software package, SPSS version 20.0 (SPSS Inc., Chicago, IL, USA). Methods used in this study were χ^2^, and analysis of variance with *post hoc* comparison of means when significance was found. Statistical significance was set at *p* < 0.05. The Kruskal–Wallis test was used for non-parametric analyses where appropriate. Multiple logistic regression analysis was used to evaluate the correlation between the factors predictive of requirement of treatment for NAS. Cox regression was performed with maternal methadone and gestational age as covariates to estimate the survival curve for onset of withdrawal requiring treatment between infants categorized by feeding modality on the first 2 days of life.

## Results

A total of 226 medical charts were reviewed. Infants born at <30 weeks gestation (*n* = 5) and those transferred to a quaternary hospital for surgery (*n* = 2) were excluded. A further 24 infants who were nil by mouth were excluded in addition to 1 infant who required treatment for withdrawal before the first feed. After exclusion, the total sample size was 194. There were a total of 150 infants in the formula group, 32 infants in the breast fed, group and 12 in the EBM group.

### Maternal characteristics

The maternal characteristics are summarized in Table 1. The EBM group had a higher methadone dose compared to all other groups (*p* = 0.03). There was no difference in the method of feeding if delivered by cesarean section. Cesarean section was shown to be a significant confounder in a previous study (10) and was therefore included in all analyses.

**Table 1:** **Maternal characteristics**.

Characteristics	Predominantly breastfed	Expressed breast milk	Formula feeding	*p* value
	*n* = 32	*n* = 12	*n* = 150	
Maternal age, median (IQR) (years)	26.5 (22–34.3)	28 (26.3–31.5)	27 (23–30)	0.72
Smoker *n* (%)	5 (16)	2 (17)	41 (27)	0.30
Polydrug^a^ *n* (%)	12 (38)	4 (33)	37 (25)	0.30
Methadone dose, median (IQR) (mg/day)	65 (47.5–100)^b^	97.5 (81.3–137.5)^b,c^	70 (45–96.3)^c^	0.03
Delivery by cesarean section *n* (%)	7 (22)	1 (9)	41 (29)	0.49

*Kruskal–Wallis test was used for non-parametric variables*.

*^a^Use of two or more different classes of addictive drugs, including methadone*.

*^b,c^Means with the same footnote are statistically significantly different from each other*.

### Infant characteristics

Table 2 outlines the infant characteristics. There were more premature infants in the EBM group and this was statistically significant compared to the breastfed group (*p* = 0.02) but not the formula fed group (*p* = 0.09). There was no statistically significant difference in average NAS scores (*p* = 0.47) or the rates of NAS requiring pharmacologic treatment between the groups (*p* = 0.11), and peak morphine dose for infants that withdrew was similar between groups (*p* = 0.2).

**Table 2:** **Characteristics and outcome of infants**.

Characteristics	Predominantly breastfed	Expressed breast milk	Formula feeding	*p* value
	*n* = 32	*n* = 12	*n* = 150	
Gestational age, median (IQR) (weeks)	39 (37.5–40)	36.6 (35–39.8)	38.5 (36.7–40)	0.14
Gestation <37 weeks *n* (%)	4 (13)^a^	6 (50)^a^	41 (27)	0.04
Male gender, *n* (%)	18 (58)	8 (67)	77 (52)	0.52
Maximum dose of morphine required, median (IQR) (mg/kg/day)	0.5 (0.5–0.7)	0.7 (0.53–0.84)	0.5 (0.5–0.7)	0.20
Average NAS score, mean ± SD	5.1 (1.3)	5.7 (0.9)	5.4 (1.1)	0.47
Required treatment for NAS *n* (%)	23 (72)	12 (100)	121 (81)	0.11

*^a^Means with the same footnote are statistically significantly different from each other*.

Multiple logistic regression was performed examining variables predictive of NAS onset requiring treatment (Table 3).

**Table 3:** **Multiple logistic regression model for variables predictive of requiring pharmacologic treatment for NAS in infants of methadone-maintained mothers**.

Variables	Odds ratio (95% confidence interval)	*p* value
Maternal methadone dose (mg/day)	1.03 (1.02–1.49)	<0.001
Gestational age at delivery (weeks)	1.27 (1.08–1.5)	0.004
Predominantly breastfed first 2 days of life	2.12 (0.8–5.73)	0.14
Polydrug use	1.35 (0.62–3.0)	0.46
Delivery by cesarean section	1.37 (0.57–3.3)	0.48

Higher maternal methadone dose (>100 mg/day) was independently associated with an increased need for pharmacologic treatment for NAS, whereas prematurity was associated with a reduced risk. In addition, when controlling for maternal age, predominant breastfeeding in the first 2 days of life was associated with a 37% reduction in need for NAS treatment. Polydrug use or delivery by Cesarean section did not significantly impact the need for pharmacotherapy.

Cox regression analysis was used to analyze variables predictive of time to onset of NAS (Table 4).

**Table 4:** **Cox regression model for variables predictive of time to onset of NAS among infants of methadone maintained mothers**.

Variables	Hazards ratio (95% CI) univariate	Hazards ratio (95% CI) multivariate	*p* value (multivariate)
High maternal methadone dose (>100 mg/day)	1.65 (1.16–2.33)	1.65 (1.15–2.36)	0.007
Gestational age <37 weeks	0.61 (0.42–0.89)	0.56 (0.38–0.83)	0.004
Predominantly breastfed first 2 days	0.70 (0.45–1.09)	0.72 (0.45–1.15)	0.04
Delivery by cesarean section	0.98 (0.68–1.42)	1.02 (0.70–1.49)	0.93

**Figure 1:**
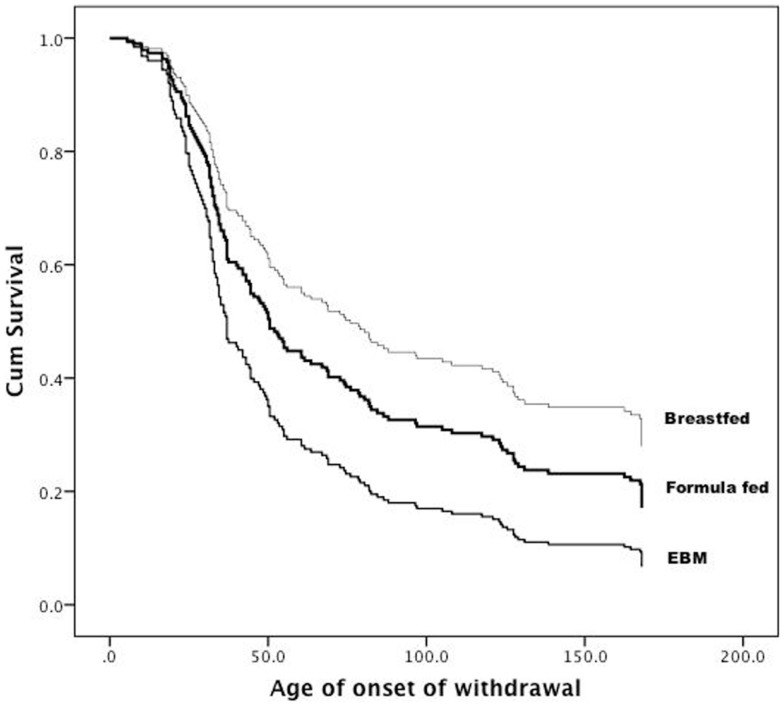
**Age (in hours) at which medications were commenced for NAS within the three groups**.

Breastfeeding during the first 2 days of life was associated with a delayed onset of NAS (*p* = 0.04). Median time to withdrawal for term infants was significantly lower compared to infants born at <37 weeks (47 vs. 75 h). Figure 1 depicts the significant trend toward the benefit of breastfeeding. The apparent benefit of formula feeding over EBM after 50 h of life cannot be over interpreted due to the small sample size of the EBM group. Similar results were obtained on adjusted analysis.

## Discussion

Infants exposed to methadone *in utero* are at risk of developing NAS (11). Breastfeeding is currently recommended by most health authorities for infants born to methadone-dependent mothers (6). Methadone-exposed mother/infant dyads are a group that could benefit substantially from the known advantages of breastfeeding, in particular, by promoting mother/infant bonding (12), alleviating infant agitation (13) and decreasing maternal stress response (14).

Jansson et al. (15) showed that the amount of methadone transmitted through breast milk is in the range of 21–462 ng/mL. To what extent this amount of methadone impacts on infants exposed *in utero* remains unclear.

Our study aimed to compare the effect of the breastfeeding process from the ingestion of human breast milk on the onset of NAS. In particular, infants who were predominantly breastfed in the first 2 days of life had a delayed onset of NAS compared to infants who were fed EBM. These results are concordant with previous studies that have shown a positive effect of breastfeeding on the outcome of NAS (4, 5). Abdel-Latif et al. (2) stated that there was no difference between infants who were fed breast milk by bottle or by gavage tube and those who were exclusively breastfed; however, no statistical data or comparisons between the groups were described.

In this study, the feeding modalities in the first 2 days were compared, to eliminate the confounder of difficulties with breastfeeding once an infant undergoes withdrawal, which introduces a significant selection bias (14).

A potential weakness in this study is the small sample size of the EBM group. It was rare to find a mother who would feed by EBM without breastfeeding or supplementing feeds with formula. Obtaining a clearer result of the effect of human breast milk alone without breastfeeding would require a randomized control trial, which would be ethically problematic.

The identification of other classes of substance misuse among our sample population is limited by maternal self-reporting. Routine urine toxicology was not performed on mothers who presented for delivery, and neonatal urine toxicology was only performed when requested. Thus, our study cannot discount the possibility of polydrug misuse as a contributing factor to NAS outcome as indicated by Abdel-Latif et al. (2).

It was surprising that feeding modality did not significantly impact the likelihood of developing NAS, which is not concordant with the findings of Abdel-Latif et al. (2) Our result suggests that despite being breastfed in the first few days of life, infants exposed to methadone *in utero* may have a similar chance of developing NAS compared to formula fed infants, but would have a considerably delayed onset. Therefore, monitoring of the infant for the first 5–7 days of life is critical to ensure that infants are not sent home before they develop more severe symptoms of NAS.

It is indeed a possibility that the more intense supportive techniques (swaddling, nursing in a quiet environment, etc.) closely associated with the act of breast feeding may help to calm and comfort a petulant NAS infant and hence reduce the withdrawal symptoms.

## Conclusion

The study concludes that breastfed infants were similarly affected by NAS as those fed by EBM or formula. We support the current concept that breastfeeding should be encouraged for all methadone-maintained women with infants at risk of NAS unless medically contraindicated. The act of breastfeeding does not have any negative impact on NAS and has additional valuable properties that should further encourage breastfeeding in this cohort of infants.

## Author Contributions

AL conceptualized and designed the study, drafted the initial manuscript, and approved the final manuscript as submitted. JJ designed the data collection methods, collected data, drafted the initial manuscript, and approved the final manuscript as submitted. AN carried out the initial analyses, reviewed and revised the manuscript, and approved the final manuscript as submitted. RN conceptualized, coordinated and supervised the manuscript preparation, critically reviewed the manuscript, and approved the final manuscript as submitted.

## Conflict of Interest Statement

The authors declare that the research was conducted in the absence of any commercial or financial relationships that could be construed as a potential conflict of interest.

## Abbreviations

EBM, expressed human breast milk; NAS, neonatal abstinence syndrome.

## References

[1] Ballard JL. Treatment of neonatal abstinence syndrome with breast milk containing methadone. J Perinat Neonatal Nurs (2002) 15:76–85. 10.1097/00005237-200203000-00008 11911622

[2] Abdel-Latif ME Pinner J Clews S Cooke F Lui K Oei J. Effects of breast milk on the severity and outcome of neonatal abstinence syndrome among infants of drug-dependent mothers. Pediatrics (2006) 117(6):1163–9. 10.1542/peds.2005-1561 16740817

[3] Malpas TJ Darlow BA Horwood J Breastfeeding reduces the severity of neonatal abstinence syndrome. J Paediatr Child Health (1997) 33:A38.

[4] Malpas TJ Darlow BA. Neonatal abstinence syndrome following abrupt cessation of breastfeeding. N Z Med J (1999) 112:12–3. 10073159

[5] Begg EJ Malpas TJ Hackett LP Ilett KF. Distribution of R- and S- methadone into human milk during multiple, medium and high oral dosing. Br J Clin Pharmacol (2001) 52:681–5. 10.1046/j.0306-5251.2001.01506.x 11736879 PMC2014565

[6] NSW Health Department Policy Directive. Neonatal Abstinence Syndrome Guidelines. (2012). Available from: http://www.health.nsw.gov.au/policies/pd/2005/PD2005_494.html

[7] Philipp BL Merewood A O’Brien S Methadone and breastfeeding: new horizons. Pediatrics (2003) 111:1429–30 10.1542/peds.111.6.1429 12777563

[8] Santoro W Jr Martinez FE Ricco RG Jorge SM. Colostrum ingested during the first day of life by exclusively breastfed healthy newborn infants. J Pediatr (2010) 156(1):29–32. 10.1016/j.jpeds.2009.07.009 19783000

[9] Finnegan L Neonatal abstinence syndrome: assessment and pharmacotherapy. In: Rubaltelli FF Granati B Granti B, editors. Neonatal Therapy: An update. New York: Elsevier Science (1986). p. 122–46.

[10] Liu A Jones M Murray H Cook C Nanan R. Perinatal risk factors for the neonatal abstinence syndrome in infants born to women on methadone maintenance therapy. Aust N Z J Obstet Gynaecol (2010) 50(3):253–8. 10.1111/j.1479-828X.2010.01168.x 20618243

[11] American Academy of Pediatrics Committee on Drugs. Transfer of drugs and other chemicals into human milk. Pediatrics (2001) 108:776–89. 10.1542/peds:2013-1985 2677964

[12] Jansson LM Velez ML Harrow C. Methadone maintenance and lactation: a review of the literature and current management guidelines. J Hum Lact (2004) 20(1):62–70. 10.1177/0890334403261027 14974702

[13] Gray L Miller LW Phillip BL Blass EM. Breastfeeding is analgesic in healthy newborns. Pediatrics (2002) 109:590–3. 10.1542/peds.109.4.590 11927701

[14] Mezzacappa E Kelsey R Katkin E. Breastfeeding, bottle feeding and maternal autonomic responses to stress. J Psychosom Res (2005) 58:351–65. 10.1016/j.jpsychores.2004.11.004 15992571

[15] Jansson LM Choo R Velez ML Harrow C Schroeder JR Shakleya DM Methadone maintenance and breastfeeding in the neonatal period. Pediatrics (2008) 121(1):106–14. 10.1542/peds.2007-1182 18166563

